# Individual and Occupational Differences in Perceived Organisational Culture of a Central Hospital in Vietnam

**DOI:** 10.1155/2018/3759290

**Published:** 2018-12-24

**Authors:** Huy Nguyen Van, Au T. H. Nguyen, Thu T. H. Nguyen, Ha T. T. Nguyen, Hien T. T. Bui, Phuong T. Tran, Anh L. T. Nguyen

**Affiliations:** ^1^Department of Health Management and Organization, Institute for Preventive Medicine and Public Health, Hanoi Medical University, 01 Ton That Tung Str., Dong Da Dist., Hanoi, Vietnam; ^2^Department of Infection Control, Quang Nam General Hospital, Tam Hiep Commune, Nui Thanh District, Quang Nam, Vietnam; ^3^Hanoi Medical University, 01 Ton That Tung Str., Dong Da Dist., Hanoi, Vietnam; ^4^Department of Health Economics, Institute for Preventive Medicine and Public Health, Hanoi Medical University, 01 Ton That Tung Str., Dong Da Dist., Hanoi, Vietnam

## Abstract

Many hospitals in developing countries, including Vietnam, are facing the challenges of increasingly noncommunicable diseases and the financial autonomy policy from the government. To adapt to this new context requires understanding and changing the current organisational culture of the hospitals. However, little has been known about this in resource-constrained healthcare settings. The objectives of this study were to examine the four characteristics of the organisational culture and test selected individual and occupational differences in the organisational culture of a Vietnam central hospital. In a cross-sectional study using the Organisation Culture Assessment Instrument (OCAI) with the Competing Value Framework (CVF), including 4 factors,* Clan, Adhocracy, Hierarchy*, and* Market*, health workers currently working at Quang Nam General Hospital were interviewed. The results indicated the current cultural model was more internally focused with two dominant cultures, Clan and Hierarchy, while, for the desired model, the Clan culture was the most expected one. Comparing between the current and desired pattern, the down trend was found for all types of culture, except the Clan culture, and there were significant differences by domains of organisational culture. Furthermore, the current and desired models were differently distributed by key individual characteristics. These differences have raised a number of interesting directions for future research. They also suggest that, to build a hospital organisational culture to suit both current and future contexts as per employees' assessment and expectation, it is important to take individual and institutional variations into account.

## 1. Introduction

Organisational culture (OC) is an anthropological metaphor used to inform research and consultancy, to explain organisational environments [1], and to consider improving organisational performance [2]. It is a system of shared values and beliefs which produces norms of behaviour and establishes an organisational way of life [3]. This term is popularly used in the business areas and called corporate culture [4]. Recently, organisational culture has been increasingly seen as crucial in healthcare system operations and quality of care [5].

A great body of research has been done to explore the effect of organisational culture on organisational performance in both high- and low-income countries. It showed a positive relation between corporate culture and better organisational commitment and financial performance of the organisation in Malaysian companies [6]. In a research on both Hongkong and Australian managers conducted in 2004, OC was found to affect job satisfaction and commitment of staffs working within an organisation [7]. The interest in OC has been gradually growing in health services research with a greater number of authors having studied about it in England, Australia, the USA, and Canada [5]. There have been an increasing number of studies focusing on the effect of OC in different areas: how OC affects nursing research utilisation in England [8], how it affects performance of organisations in acute English hospitals [9], and how clinicians utilise clinical information systems in an Australian hospital [10] or discover the relationship between OC and leadership behaviour and job satisfaction among Taiwan hospital nurses [11].

With the importantly recognised role of OC to an organisation [11], many instruments have been developed to measure it. Among the approaches to examine the OC, the Organisation Culture Assessment Instrument (OCAI) using the Competing Value Framework (CVF) is a popular method and has been widely applied in prior research up to date. This OCAI has also been validated in healthcare contexts before [12]. A number of empirical studies have confirmed the CVF as an effective tool for evaluating organisational culture such as Linnenluecke and Griffiths in 2010 [13], Cameron and Quinn in 2006 [14], Howard in 1998 [15], and Lamond in 2003 [16]. Since then, CVF has become a common model in OC research and is a useful tool to study and initiate cultural change to achieve expected outcomes [17].

**Figure 1:**
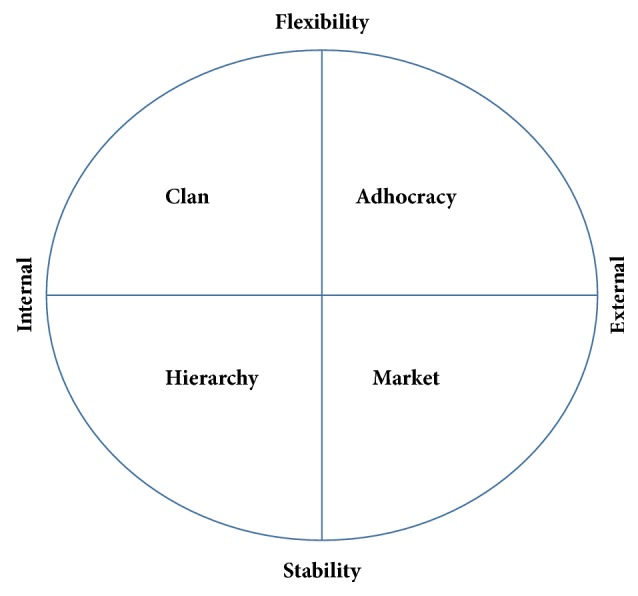
Factor structure of the OCAI reflective of the Competing Values Framework, adapted from Cameron and Quinn [14].

According to the OCAI, culture is assessed based on core values, shared assumptions, and common approaches to work. The instrument uses four factors in the CVF to classify culture as the synchronisation of two among these factors: stability versus flexibility in work approaches and internal versus external focus of the organisation [12], as presented in Figure 1.

Figure 1 describes four different types of OC [14]. The first is the* Clan culture*, the upper left corner, which includes flexibility and internal focus. It is considered to be representative of a family-style organisation, wherein members of the organisation are involved in decision-making and teamwork is an important aspect of work. The second is the* Adhocracy culture*, which is delineated by the flexibility and external focus aspects, and implies innovation as a means of organisational functioning. One of the aspects of Adhocracy is its emphasis on specialisation and rapid change within the organisation. The third type is the* Hierarchy culture* which is delineated by the internal focus and stability aspects. It is concerned largely with stability in organisational functioning and has clear guidelines regarding the manner in which an organisation should approach. It is typified by a vertical approach to the levels in the organisational hierarchy and focuses largely on smooth-running efficiency. The final type is the* Market culture* which is delineated by the external focus and stability aspects. This aspect of culture is concerned largely with competitiveness and winning. The Market culture is driven by the need to create transactions with external bodies as a means of gaining an advantage in their organisational niche.

The OCAI has been applied in quite few studies, but only a limited number of them focused on the health setting. Recent studies have tended to explore the relationship between organisational culture and hospital performance, job satisfaction, or human resources practice [9, 18]. Few studies have confirmed that managers' assessment of organisational culture differs from those of nonmanagers [18], so whether there are differences in perceived organisational culture between clinical and nonclinical staffs remains not fully investigated. In Vietnam, up to date, only one study has been undertaken to apply the OCAI in measuring organisational culture in the banking environment [19]; little research on OC using other tools or none has been conducted in the health sector. The objectives of this study were to examine the key characteristics of organisational culture and test selected individual and occupational differences in the organisational culture of a central hospital in Vietnam. This study is important given the current context featuring that many hospitals in developing countries, including Vietnam, have to respond to a dramatic change in the disease pattern and health policies. In many of such countries, communicable diseases remain high and noncommunicable diseases are increasing over time, while the financial autonomy policy required for a hospital system has been introduced by the government.

## 2. Methods

### 2.1. Research Design and Location

A cross-sectional quantitative study using the structured questionnaire to interview with respondents has been carried out at a central hospital of Vietnam, Quang Nam General Hospital. Quang Nam is a province in the South-Central Coast region of Vietnam, located in the middle of a north-south traffic axis. About 1.5 million people live in the province of Quang Nam and the natural area is 10,406.83 km^2^ [20]. According to the Vietnamese hospital classification, among the four classes, the first one is ranked as the best in terms of healthcare quality. Under this ranking, the Quang Nam General Hospital province is a first-class hospital which serves largely medical examination and treatment for clinical health conditions. The hospital is currently composed of 20 clinic departments, 8 subclinical departments, and 7 function rooms. 701 health workers participate in hospital activities. The healthcare facility counts up 825 beds out of a planned capacity of 600. There, the youth, enthusiastic, and energetic staffs work in a spacious and modern environment [21].

### 2.2. Participants, Sample Size, and Participants

In total, 566 health workers currently working at Quang Nam General Hospital participated in our study.

### 2.3. Measures

The main indicator of the current study was the organisational culture scale measured with four domains,* Clan, Adhocracy, Hierarchy*, and* Market*, which were assessed using the OCAI. The OCAI is based on the theory about the CVF, which was developed by American researchers Cameron and Quinn in 2006 [22]. The purpose of this theory is to help the understanding of organisational phenomena, such as organisational design, stages of life cycle development, organisational quality, leadership roles, and management skills. Organisations are functioning in fast changing environments, which requires them to adapt constantly. That is the reason why the organisation should be able to define the direction it wants to take in the future. The OCAI method assesses six key aspects of the organisational culture (*dominant characteristics*,* organisational leadership*,* management of employees*,* organisational glue*,* strategic emphases*, and* criteria of success*). For each of these aspects, four statements defining four types of organisations were proposed to the participants. As per the participants' responses, what the organisation currently is and what organisation they are expecting in the future could be defined. For instance, the following statement could help to define how clannishly the organisation is shaped (Clan scale): “The organisation is a very personal place. It is like an extended family. People seem to share a lot of them.” Besides, the affirmation “The organisation is a very dynamic and entrepreneurial place. People are willing to stick their necks out and take risks” defines an adhocracy organisation, according to the Adhocracy scale. An example of a statement for the hierarchy organisation (Hierarchy scale) would be “The organisation is a very controlled and structured place. Formal procedures generally govern what people do.” And a market organisation (Market scale) can be defined as follows: “The organisation is very results-oriented. A major concern is with getting the job done. People are very competitive and achievement-oriented.”

For each of the six aspects, participants of the survey were asked to rank the 4 statements (*Clan*,* Hierarchy*,* Adhocracy*, and* Market*) according to the vision of their organisation. Every participant was asked to distribute between the four statements an amount of 100 points, by weighting each item. By dividing 100 points over 4 statements, respondents have to weight and choose—just like the CVF or reality. This method of weighting differs from the ordinary Likert scale, in which the respondents are usually asked to evaluate options on a scale from 1 to 5, or 1 to 7, from "completely disagree" to "completely agree." In their study, Cameron and Quinn used both types of scale. However, the constant rating scale has advantages over the Likert scale as follows: (i) the use of an ipsative rating scale differs from the Likert scale, which highlights the differences in organisational culture; (ii) when using the Likert scale, the respondents tend to rate all answers as high or low, while the total constant scale urges the respondents to promptly trade to choose what is really the existence in the organisation.

At each choice of the 24 items (6 aspects and 4 statements for each), the survey respondents were given the selection points in two columns. Points mentioned in the column "*Now*" correspond to the perception of the organisation that the respondent currently has, while those mentioned in the "Preferred" column correspond to his/her expectations of the organisation in the future. In order to average the OCAI scores, the first step was to add together all A responses in the "*Now*" column and divide the total by 6. This was to compute an average score for the A alternatives in the "*Now*" column. After that, we repeated this computation for the remaining alternatives as well as the "*Preferred*" column. The second step was to add all A responses in the "*Preferred*" column and divide by 6. In other words, compute an average score for the A alternatives in the "*Preferred*" column. Next, add together all B responses and divide by 6. Repeat this computation for the C and D alternatives. According to Cameron and Quinn, the score for Alternative A represents the Clan culture, the score of Alternative B the culture of Adhocracy, the score of Alternative C the culture of Market, and the score of Alternative D the culture of Hierarchy. This method determines the combination of the four types of culture that exist in an organisation. The results are displayed on a chart that shows the difference between the current and preferred cultures.

The scale reliability for each of the four archetypal profiles has been demonstrated as sufficient, with Cronbach's alphas ranging from 0.70 to 0.80 [23, 24]. In this study, we also conducted a pilot study to verify the suitability of the OCAI. Pilot research was conducted on 30 hospital health staffs. The psychometric tests showed that Cronbach's alpha of the Clan, Adhocracy, Market, and Hierarchy culture scale for the "Now" column was 0.636, 0.601, 0.692, and 0.621, respectively, and, for the "Preferred" column, Cronbach's alpha of the Clan, Adhocracy, Market, and Hierarchy culture scale was 0.686, 0.666, 0.692, and 0.621, respectively. Although Cronbach's alpha of some domains was slightly lower than expected (<0.70), it is quite acceptable in research on social sciences such as organisational culture. In terms of face validity, respondents were able to understand all the survey questions during the pilot. The above results showed therefore that the instrument was technically feasible.

The demographic variables include age (under or over 30 years), gender (male or female), marital status (single or married), educational level (intermediate, college, or university level), job tenure (under 2 years, 2 years to 5 years, or over 5 years), position (manager, administrator, technician, or clinical provider), and workplace (functional department, clinic, or laboratory). They were measured using single items for prospective inclusion as control variables in the analysis.

### 2.4. Data Collection and Research Ethics

The study protocol was approved by the scientific panel from the Department of Health Management and the Institute for Preventive Medicine and Public Health, Hanoi Medical University, in Vietnam. Participation of all respondents was anonymous and voluntary. As process, all 701 health workers working at Quang Nam General Hospital were informed about the research content and objectives as well as how the interview data would be documented and reported and that their confidentiality would be respected and they could withdraw at any time. In total, 566 employees provided verbal informed consent and completed the survey interviews, making up a response rate of 80.7%. Most of the respondents who did not participate in the survey revealed that they had maternal or holiday leaves, took further study, or executed other missions as assigned by hospital leaders/managers. This data collection was taken by a Master of Hospital Management who has been experienced in healthcare system-based research. She was also one of the investigators who jointly designed the research proposal and conducted the research. As she is currently working at this hospital, she is familiar with the procedure and the process of data collection therefore ran smoothly.

### 2.5. Data Analysis

Data were analysed using STATA 10.0. Descriptive statistics such as mean, median, standard deviation, frequency, and percentage were used to describe the organisation culture. Means and standard deviations were used when data was normally distributed. As each respondent allocated a score of 100 over four types of responses for the A (Clan), B (Adhocracy), C (Market), and D (Hierarchy) culture, the minimum and maximum scores for each type were 0 and 100, respectively. The* t*-test was applied to assess mean differences between two groups and one-way ANOVA to serve comparing more than two means. The value of* p*<0.05 was set as statistically significant. We utilised these tests to be in line with the research objective which was to test mean differences in, rather than to examine factors associated with, the outcome.

## 3. Results

### 3.1. The Selected Sociodemographic Characteristics of the Sample

The key characteristics of the respondents are displayed in Table 1. Among 566 respondents involved in the survey, most (72.4%) were female. The mean age of the respondents was 31.9 years. The majority of the respondents were over 30 years old (57.8%) and married (80.7%). Regarding education levels, 39.4 % of the responders completed intermediate level, followed by college level (33.6 %) and university level (27%). The proportions of managers and staffs were 10.3% and 90.7%, respectively. The average years of working were 7.7 and most of the respondents reported having 5 years or more of working time. In terms of workplace, while more than half of the staffs were working inside the labs, the figure for those in the functional department and clinic was 17.1% and 30.6%, respectively.

### 3.2. The Organisational Culture of Quang Nam General Central Hospital as Assessed by the Respondents

The present and desired organisational culture patterns of Quang Nam General Central Hospital as assessed by its workforce are shown in Table 2. The organisational culture pattern was displayed in four main types: Clan, Adhocracy, Market, and Hierarchy. The two types of culture that mainly prevailed at present were Clan and Hierarchy with relative mean scores of 26.7 and 26.6, respectively. The desired model of organisational culture also followed the same pattern as the Clan and Hierarchy culture shared more parts compared to the other types of culture. The differences between the current and preferred culture suggested that there was a decline in the mean score for all types of culture, except Clan which increased significantly by 4.8%.

The principal characteristics of the current and desired organisational culture by domains are presented in Table 3. In general, there were differences in all domains of organisational culture, namely, dominant characteristic, organisational leadership, management of employees, organisational glue, strategic emphases, and criteria of success, between the current and the preferred culture. By comparing between the current and preferred situation, the Clan score tended to increase significantly in all types of organisational culture in each of the domains (*p*<0.05). By contrast, the score of the remaining types of culture (Adhocracy, Hierarchy, and Market) declined remarkably (*p*<0.05 in most cases).

### 3.3. Individual and Occupational Differences in the Organisational Culture of a Central Hospital

Table 4 examines the current organisational culture by key individual characteristics. There were statistically significant differences in different cultures by key personal and occupational characteristics. As observed, staffs gave very low scores for the Market culture and high scores for the other three types of culture. Meanwhile, managers were in favour of the Clan and Hierarchy culture but they were not keen on the Adhocracy culture like their staffs. While both males and females scored high for the Clan and Hierarchy culture, males had a tendency to give higher scores for the Adhocracy culture. Hierarchy was the most desirable culture for respondents working for management-administration-technique-service (M-A-T-S) followed by the Clan and Adhocracy culture. They also reported higher scores for Adhocracy than those in treatment and paraclinical areas. Respondents with other ethos revealed higher scores for the Clan and Hierarchy culture.

Table 5 examines the desired organisational culture by key individual characteristics. In terms of position, both managers and staffs desired the highest score for the Clan culture and the lowest score for the Market culture. However, staffs desired a higher score for the Clan and Adhocracy culture than managers and managers gave a higher score for the Market culture than their counterparts. Both males and females desired the highest score for the Clan culture; however, males preferred the Adhocracy culture. In terms of age, both age groups expected to have higher Clan culture in the workplace; yet, those aged more than 30 years wanted more of the Adhocracy and Market culture than their younger counterparts. Both college and university respondents desired the highest score for the Clan culture; however, university respondents desired a higher score for the Adhocracy culture than college ones. Participants working in M-A-T-S scored higher for the Clan and Adhocracy culture than those serving in clinical and paraclinical areas. By contrast, M-A-T-S respondents scored much lower for the Market culture than those serving in clinical and paraclinical areas. While both married and single respondents reported the highest desire for the Clan culture and the lowest desire for the Market culture, single respondents gave a higher score for the Adhocracy culture. All of the above observed differences were significant (*p*<0.01).

## 4. Discussion

### 4.1. The Organisational Culture of a Central Hospital in Vietnam

The present pattern of organisational culture of Quang Nam General Central Hospital did not feature as a specific type but was the combination of all four cultural types, of which two cultural trends were significantly predominant than the others: the Clan culture had a total of 26.7/100 points and the Hierarchy culture had 26.6/100 points. This pattern showed that the organisation was more internally focused, which highly valued the importance of internal stability rather than building the organisation's position in a competitive context. This culture model is commonly seen in state organisations that are subsidised by the government. This study's result is similar to those indicated by Ping Zhou et al. (2011) when they studied organisational culture at 87 public hospitals in China in preparation for reform in public health (similar to the current context of the Vietnamese health). In this study, Zhou also confirmed the organisational culture of public hospitals in China was mainly internally focused [18]. In terms of the desired pattern of organisational culture, the results appeared to follow the same pattern as it was also more internally focused as the Clan and Hierarchy culture had higher scores; particularly the Clan culture accounted for 31.5/100 points. Our study coincides with the US study of Bing-You and Varaklis (2016) at the US Department of Health and Education, which indicated that the organisational cultural trend preferred by the participants in the survey was the Clan culture [25].

There are two most significant differences when comparing the present organisational culture and the desired organisational culture of Quang Nam General Hospital's staff. Firstly, the score for the Clan culture in the future increased by 4.8 points compared to the present, indicating that the health workers wish that the hospital created a more cohesive and supportive working environment. Therefore, the staffs could consider the hospital as a big family in which people shared the values and common goals and expected to be empowered more by their leader acting as a mentor or a father in the family. This pattern helps to consolidate staffs' commitment to the hospital with loyalty and colleagues. The second difference was that the desired Market culture is 2.7 points lower than the present pattern, which may be due to the current policy shifting from a subsidised to a self-reliant hospital that health workers were feeling pressured under the Market culture. They were concerned not only about their profession but also about the profitability of their work while the price structure of health services is still somewhat illogical nowadays. They wish that the leaders understood the needs and expectations of their staffs in order to explain and motivate them and create rational internal policies which stimulate employees to work and improve the competitiveness of the hospital. Moreover, it is necessary that the managers integrate more strategic and visionary solutions to enhance the competitiveness and capability of the hospital, especially when Vietnam's health sector is transforming towards full autonomy and global integration.

Regarding each individual variable in the six components of organisational culture, dominant characteristics of organisation, organisational leadership, management of employees, organisational glue, strategic emphases, and criteria of success, we found that all 6 components of the current culture differed from those of the desired culture (there is no conflict between the measurement variables). It has been noticed that the difference in the score of all components in the hospital's current culture pattern was insignificant, indicating the internal and control focus as the general model. Meanwhile, the future pattern was almost identical with two main aspirations: to increase the family aspect and to reduce the Market aspect of the culture. This consistency was praised by Cameron and Quinn in a study of 334 colleges and universities in the United States; managers considered that there would be no inconsistencies influencing the performance of the organisation [14]. Consistency also helped managers to determine the exact model that they needed to build in a certain direction. The analysis of each variable also helped to determine that two components in urgent need to change are organisational glue and strategic emphases of the hospital. Of the six variables that made up the organisational culture model, these two variables had the greatest difference between the present and the future, which meant that the adjustment of these components was required to reach the desired organisation.

### 4.2. Individual and Occupational Differences in the Organisational Culture of a Central Hospital

The analysis of organisational culture assessed by different groups of health staffs would help managers determine whether there are differences in the mindset of groups of health staffs. Based on that, the leader could develop the most appropriate policies which focused on specific groups in order to achieve consistency in the desired organisational culture pattern. It also suggests the leaders to manage human resources overall and/or by groups effectively.

Regarding the current organisational culture, Quang Nam General Hospital was homogenised with internal and control focus, which formulates two dominant cultural trends: the Clan and Hierarchy culture. There were a number of differences in assessment among health staff about the current organisational culture. Firstly, with regard to the job position, managers evaluated the Clan culture as the most dominant, with a higher score compared to that of the employees, while the most prevalent domain rated by the employees was the Hierarchy culture, with higher scores than managers' assessment. This implies that managers consider the current culture as more intimate and sharing than employees did, while employees perceive the current culture model as the Hierarchy culture with the clear rules rather than friendliness. Our findings are in concert with the study of Ping Zhou et al. on health workers and patients of 87 public hospitals in China in 2009 and Phuong's research on the staff at Sacombank in Vietnam in 2013, indicating that managers often assessed the current friendliness in an organisation higher while the staffs tended to rate the hierarchy higher [18, 19].

There was no significant difference in assessment of organisational culture among different groups of health staffs in terms of gender, age, educational level, seniority, and marital status. This consistency showed that the current organisational culture of the hospital was clearly demonstrated. However, there was a significant difference in the assessment of organisational culture among staffs at different positions, which mirrors the finding from 87 public hospitals in the study conducted by Ping Zhou et al. that “the most relevant factor for culture assessment is the field of activity” [18]. People who worked in the clinical and paraclinical department tended to score the Clan and Market culture significantly higher than the other two and higher than the remaining groups did for these cultures. This could be explained by the nature of work of the clinical job that requires working in a team and direct contact with patients so this group feels intimate and shared in the work. In addition, due to direct income generation, they directly recognised the pressure of the Market culture trend. By contrast, the administrative group working in the functional divisions gave higher scores to the Hierarchy and Adhocracy culture than clinical and paraclinical groups. Because of the particularity of administrative and managerial work, the group felt more of the Hierarchy culture while lacking intimacy and sharing among team members.

In terms of the desired organisational culture, the patterns selected by different groups of health workers show the uniformity in the trend of increasing the Clan and reducing the Market focus. This would make it easier for managers to identify the organisational culture that needs to be adapted in the future. Notwithstanding this consistency, there are some disparities in the desired organisational culture among different individual characteristics and, by understanding these differences, the managers could be better equipped to define the policies that have great impacts on each target group of health workers. The first difference was that even though the hospital managers and staffs expected the future hospital culture to be dominated by the Clan culture which is indicated by the highest scores, employees had higher expectation for this culture than the managers did. On the other hand, managers expected to have more of the Market culture than the employees' group. This indicated that managers wanted the organisational culture to be more competitive and emphasised efficiency more. It is naturally and socially acceptable for managers to lean toward the Market culture so as to respond to a transitioning economy as Vietnam as well as to a situation where they have limited choice to finance their health activities when the government allocates a small budget for healthcare today. However, Vuong argues [26] that as science can make a significant impact on the economy of developing countries such as Vietnam, it is important for us—whoever we are—to recognise and take full advantage of it and its costs must be taken into account. Secondly, in the desired organisational culture model, the group of male, single, and administrative staffs expected the dominance of the Adhocracy culture much higher than the group of female, married, and clinical staffs. This result has a practical meaning for managers who want to advance a new policy in the organisation as the group of male, single, and administrative staffs are more willing to look for creativity and innovation and could be the main driving force for the positive changes within the organisation. Additionally, regarding the fields of working, the fact that the administrative group expected the Clan culture trend in the future to be more dominant than the clinical staff group did meant that they wanted to improve the internal solidarity in a sharing working environment.

The study findings should be interpreted in light of several limitations. First, as the OC in hospital is a sensitive area in its nature, health workers would be more likely to under- or overreport their responses. Another concern is related to a measure of OC by self-report by respondents. Because of this, we designed our survey carefully in a manner in which the survey was OCAI based and anonymous which was believed to enhance the internal validity of the result. Also, due to our limited access to funding, we just approached healthcare providers such as health workers to investigate the OC. We suggest that future research take healthcare users into account in order to complement better understanding of this phenomenon.

## 5. Conclusions and Implications

The current organisational culture of the hospital is a combination of four types of organisational culture, namely, the Clan, Hierarchy, Adhocracy, and Market culture. The current cultural model is more internally focused with two dominant cultural trends, the Clan and Hierarchy culture, while, in the desired one, the Clan culture is the most expected. The expectations expressed by staffs are to increase the intimacy and trust in the Clan culture and to reduce the pressure of competitiveness and profitability from the Market culture. Differences in the current and desired organisational culture are also the most evident among groups with different job positions and work units. Managers and administrators gave lower score for the current Clan culture compared to the clinical and paraclinical groups. The desired organisational culture model desired by administrative staffs working in functional departments has more focus on the aspect of the Clan and Adhocracy culture, while the desire for the Market culture of the clinical and paraclinical group is higher than that of the administrative group. It appears that managers want the future organisational culture model to be more Market-oriented than their staff, while the staff prefer the future model to be more Clan-oriented than the managers. This preference of the Market culture by managers is understandable given a recent change in financing policies in that even public hospitals have to be financially self-reliant. Accordingly, hospital managers and leaders have to seek to sustain their healthcare. One of the ways would be to create a vision and a culture to support Market value rather than other values. This result is quite in line with behaviour economics as indicated in Vuong's study [27]. Those differences in the assessment of organisational culture of health workers in different job positions could also be due to the fact that the communication flow within the hospital is not so good that the health staffs do not clearly and thoroughly understand the strategy that the hospital has been pursuing.

The study suggests that, to build a hospital organisational culture to better suit both current and future expectations of health workers, it is important to take individual and institutional variations into account. To be specific, because one size cannot fit all, it is necessary to integrate the other cultural types into the dominant one and vice versa in order to serve both the diversity and the expectation of health staffs. In the short term, it is necessary to enhance the internal communication in order to ensure that the information has been synchronised so that all employees could understand it completely and, thereby, increase their commitment towards the hospital. In addition, the diversity of the opinions among hospital staffs requires the directors and managers to develop appropriate compensation policies to encourage their employees. In the long term, to change a hospital culture effectively, it is recommended that managers or administrators develop a long-term plan in a manner that can meet the staff's expectations of both the current and future culture.

## Figures and Tables

**Table 1:** The key characteristics of the respondents.

**Variables (n=566)**	**Frequency (**%**)**	**Mean ± SD**
***Gender***		
Male	156(27.6)	
Female	410(72.4)	
***Age***		31.9 ± 11.0
<30	239(42.2)	
>=30	327(57.8)	
***Marital status***		
Married	457(80.7)	
Single	109(19.3)	
***Educational level***		
Intermediate	223(39.4)	
College	190(33.6)	
University	153(27.0)	
***Position***		
Manager	58(10.3)	
Staff	508(89.7)	
***Seniority of working***		7.7±5.8
<2 years	17(3.0)	
2 -<5 years	156(27.6)	
>= 5 years	393(69.4)	
**Fields of working **		
Management, administration	98(17.3)	
Technique	142(25.1)	
Treatment, care	326(57.6)	
***Workplace (departments)***		
Functional	97(17.1)	
Clinic	173(30.6)	
Laboratory	296(52.3)	

**Table 2:** The characteristics of hospital organisational culture.

**Types of culture**	**Now**	**Preferred**	**Difference**	***p *(Student's *t*-test)**
**(n = 718)**	**(medium ± SD)**	**(medium ± SD)**		
Clan	26.7±8.1	31.5±7.8	4.8	**∗** **∗** **∗**
Adhocracy	24.1±5.5	23.8±6.1	-0.3	**NS**
Market	22.6±7.7	19.9±6.4	-2.7	**∗** **∗** **∗**
Hierarchy	26.6±6.2	24.8±6.8	-1.8	**∗** **∗** **∗**

*∗p* < 0.05; *∗∗p* < 0.01; *∗∗∗p* < 0.001; NS = not significant.

**Table 3:** The characteristics of hospital organisational culture by domains.

**Organisational culture by key classifications**	**Now**	**Preferred**	**Difference**	***p* (Student's t-test)**
**Dominant characteristic **				
Clan	29.0±11.8	33.3±13.0	4.3	*∗∗∗*
Adhocracy	22.6±8.9	23.9±10.8	1.3	*∗∗*
Hierarchy	23.6±9.8	19.9±9.7	-3.7	*∗∗∗*
Market	24.6±9.5	22.9±12.3	-1.7	*∗∗∗*
**Organisational leadership**				
Clan	26.8±11.2	29.3±11.5	2.5	*∗∗∗*
Adhocracy	23.6±8.7	24.5±9.3	0.9	NS
Hierarchy	24.5±8.4	22.7±10.6	-1.8	*∗∗∗*
Market	25.1±10.6	23.5±10.3	-1.7	*∗∗∗*
**Management of employees**				
Clan	26.8±12.1	32.0±13.4	5.2	*∗∗∗*
Adhocracy	22.6±10.0	22.2±9.8	-0.4	NS
Hierarchy	22.4±12.1	18.1±9.5	-4.3	*∗∗∗*
Market	27.6±12.2	27.7±12.5	0.1	NS
**Organisational glue **				
Clan	23.8±12.0	30.7±12.0	7.1	*∗∗∗*
Adhocracy	24.3±9.4	24.1±9.6	-0.2	NS
Hierarchy	25.4±12.1	19.9±9.1	-5.5	*∗∗∗*
Market	26.3±10.2	25.3±11.9	-1.0	*∗∗∗*
**Strategic emphases **				
Clan	26.5±12.9	33.0±12.8	6.5	*∗∗∗*
Adhocracy	24.4±9.7	23.1±9.9	-1.3	*∗∗*
Hierarchy	20.3±9.4	19.4±10.8	-0.9	NS
Market	28.4±11.7	24.5±11.9	-3.9	*∗∗∗*
**Criteria of success **				
Clan	26.7±11.1	30.9±12.0	4.2	*∗∗∗*
Adhocracy	26.6±10.9	24.9±9.2	-1.7	*∗*
Hierarchy	18.8±9.6	19.1±11.1	0.3	NS
Market	27.5±11.5	25.1±11.5	-2.4	*∗∗∗*

*∗p* < 0.05; *∗∗p* < 0.01; *∗∗∗p* < 0.001; NS = not significant.

**Table 4:** The characteristics of the current organisational culture by selected individual characteristics.

**Type of organisational culture**	**Clan culture**	**Adhocracy culture**	**Market culture**	**Hierarchy culture**	***p *(ANOVA test)**
**Position**					
Manager	26.7±8.3	23.9±5.6	22.6±5.8	26.4±6.2	*∗∗*
Staff	25.3±5.9	25.1±4.9	21.7±4.8	27.6±6.4	
**Gender**					
Male	26.1±8.1	24.7±5.4	22.0±5.9	26.8±6.7	*∗∗*
Female	26.7±8.1	23.7±5.6	22.7±5.9	26.5±6.0	
**Age**					
<=30	26.6±8.8	24.1±5.5	22.4±6.2	26.4±6.4	*∗∗*
>30	26.5±6.9	24.0±5.5	22.6±5.0	26.8±6.0	
**Educational level**					
College	26.9±8.2	23.9±5.4	22.6±5.8	26.3±5.9	*∗∗*
University	25.7±7.8	24.4±5.8	22.1±5.5	27.3±6.9	
**Occupational area**					
Treatment, paraclinical	26.7±7.9	23.8±5.2	22.8±5.8	26.4±6.1	*∗∗*
M-A-T-S^¥^	25.8±8.9	24.8±6.6	21.1±5.2	28.3±6.4	
**Seniority**					
< 5 years	26.8±7.4	23.7±5.7	22.6±5.7	26.4±6.4	*∗∗*
>= 5 years	26.5±8.4	24.1±5.4	22.4±5.8	26.6±6.2	
**Marital status**					
Married	26.6±8.2	24.0±5.5	22.5±5.9	26.6±6.0	*∗∗*
Single	26.4±7.8	24.2±5.5	22.3±4.9	26.4±6.0	

^*¥*^M-A-T-S: manager-administration-technical-service.

*∗p* < 0.05; *∗∗p* < 0.01; *∗∗∗p* < 0.001.

**Table 5:** The characteristics of the desired organisational culture by selected individual characteristics.

**Type of organisational culture**	**Clan culture**	**Adhocracy culture**	**Market culture**	**Hierarchy culture**	***p* (ANOVA test)**
**Position**					
Manager	31.4±7.9	23.7±5.9	20.1±6.4	24.8±6.9	*∗∗*
Staff	32.2±6.8	24.4±7.3	18.1±6.1	24.7±6.5	
**Gender**					
Male	31.2±6.6	24.6±6.8	19.5±6.5	24.6±7.3	*∗∗*
Female	31.6±8.3	23.5±5.8	20.0±6.3	24.9±6.6	
**Age**					
<=30	31.8±8.4	23.4±6.4	19.5±6.8	25.0±7.0	*∗∗*
>30	31.2±7.0	24.3±5.6	20.4± 5.8	24.1±6.5	
**Educational level**					
College	31.6±8.1	23.5±5.9	19.9±6.4	24.9±6.7	*∗∗*
University	31.4±7.2	24.5±6.6	19.4±6.4	24.7±7.1	
**Occupational area**					
Treatment, paraclinical	31.3±7.8	23.6±5.5	20.3±6.3	24.6±6.6	*∗∗*
M-A-T-S^¥^	32.5±7.7	24.7±8.3	18.1±6.5	24.7±7.8	
**Seniority**					
< 5 years	31.3±7.4	24.1±6.1	20.0±6.1	24.6±6.8	*∗∗*
>= 5 years	31.6±8.0	23.6±6.1	19.6±6.5	24.9±6.8	
**Marital status**					
Married	31.5±7.9	23.5±6.1	19.9±6.6	24.9±6.9	*∗∗*
Single	31.6±7.5	25.2±5.9	19.8±5.6	24.3±6.4	

^*¥*^M-A-T-S: manager-administration-technical-service.

*∗p* < 0.05; *∗∗p* < 0.01; *∗∗∗p* < 0.001.

## Data Availability

The data used to support the findings of this study are available from the corresponding author upon request.
